# Independent effects of phytase and β-mannanase on growth performance and nutrient utilization in broilers fed phosphorus-deficient diets without supplemental inorganic phosphorus

**DOI:** 10.14202/vetworld.2026.4133-4142

**Published:** 2026-09-18

**Authors:** Tanachot Pengsawad, Theerawit Poeikhampha, Chaiyapoom Bunchasak, Choawit Rakangthong, Sombat Prasongsook

**Affiliations:** 1 Department of Animal Science, Faculty of Agriculture, Kasetsart University, Bangkok 10900, Thailand.

**Keywords:** β-mannanase, broiler, calcium digestibility, ileal digestible energy, non-phytate phosphorus, nutrient utilization, phosphorus restriction, phytase

## Abstract

**Background and Aim:**

Phytase and β-mannanase are widely used in poultry diets to improve nutrient utilization; however, their individual and interactive effects under phosphorus (P) restriction without supplemental inorganic P remain insufficiently characterized. This study evaluated the effects of phytase and β-mannanase, alone and in combination, on growth performance, ileal digestible energy (IDE), and apparent ileal calcium (Ca) and P digestibility in broilers fed a P-deficient diet without supplemental inorganic P.

**Materials and Methods:**

A total of 720 one-day-old Ross 308 male broilers were randomly allocated to four dietary treatments in a 2 × 2 factorial arrangement comprising phytase at 0 or 1,000 FTU/kg and β-mannanase at 0 or 800 Thermostable mannanase unit/kg. Each treatment included 12 replicate pens of 15 birds. From days 8 to 24, birds received corn–soybean meal-based grower diets containing 0.40% total P, 0.14% non-phytate P, and 0.26% phytate P without supplemental inorganic P. Growth performance was evaluated, and ileal digesta were collected on day 24 to determine IDE and apparent ileal Ca and P digestibility.

**Results:**

Phytase supplementation increased final body weight by 14.4%, average daily gain by 17.8%, and feed intake by 14.4% and improved feed conversion ratio by 2.9% (p < 0.01). Phytase also increased IDE from 2,641.84 to 2,686.30 kcal/kg, Ca digestibility from 57.82% to 58.83%, and P digestibility from 71.59% to 74.83% (p < 0.01). β-Mannanase increased IDE from 2,647.01 to 2,681.13 kcal/kg (p < 0.01) and Ca digestibility from 58.00% to 58.70% (p < 0.05) but did not significantly affect growth performance or P digestibility. No significant phytase × β-mannanase interactions were detected for growth performance, IDE, or mineral digestibility, although an interaction was observed for flock uniformity (p = 0.011).

**Conclusion:**

Under the P-restricted dietary conditions, phytase primarily improved growth performance and mineral utilization, whereas β-mannanase independently enhanced energy utilization and modestly improved Ca digestibility. The absence of synergistic effects indicates distinct enzyme responses under these dietary conditions. These findings support phytase as a nutritional strategy for substantially reducing supplemental inorganic P in carefully formulated broiler diets, although longer-term validation under commercial feeding conditions is warranted.

## INTRODUCTION

Poultry production plays an important role in meeting the growing demand for high-quality animal protein, while efficient nutrient utilization is essential for improving production efficiency and environmental sustainability. However, anti-nutritional factors can limit the nutritional value of plant-based feed ingredients by reducing nutrient availability in broilers [1]. Phytate, the principal storage form of phosphorus (P) in cereals and oilseed meals, is less available to poultry and can form complexes with proteins and minerals, particularly calcium (Ca), thereby reducing nutrient digestibility and utilization [2, 3, 4]. Exogenous phytase is therefore widely incorporated into poultry diets to hydrolyze phytate, release phytate-bound P, and improve P and Ca utilization [5]. Another relevant anti-nutritional component of plant-based diets is β-mannan, a non-starch polysaccharide present in soybean meal that may interfere with nutrient utilization and induce a feed-induced immune response [6]. Dietary supplementation with β-mannanase hydrolyzes β-mannan and may alleviate these anti-nutritional effects, thereby improving nutrient and energy utilization in broilers [7, 8].

Phytase and β-mannanase act on different anti-nutritional components of plant-based diets and may therefore exert complementary effects on nutrient utilization. Phytase primarily improves the availability of phytate-bound P and associated minerals, whereas β-mannanase targets β-mannan-containing components of the feed matrix. Consequently, β-mannanase-mediated modification of plant cell-wall structures and digesta characteristics may improve nutrient accessibility and potentially influence the response to phytase supplementation. Understanding whether these enzymes exert independent, additive, or interactive effects is particularly relevant when diets are formulated under substantial nutrient restriction, where the identity and magnitude of the primary limiting nutrient may influence the response to exogenous enzymes.

Despite the well-established individual effects of phytase and β-mannanase in broiler nutrition, their combined effects under P-restricted dietary conditions remain insufficiently characterized. Previous studies have commonly evaluated these enzymes in diets containing supplemental inorganic P or as components of multi-enzyme supplementation strategies, making it difficult to distinguish their individual contributions and determine whether β-mannanase modifies the nutritional response to phytase. In particular, limited information is available on the simultaneous use of phytase and β-mannanase in grower-phase Ross 308 broilers receiving diets completely devoid of supplemental inorganic P. Under these conditions, phytate-bound P constitutes an important dietary P source, while β-mannan remains a potential constraint on nutrient and energy utilization. Therefore, evaluating both enzymes within a factorial experimental framework is necessary to distinguish their independent effects from their potential interactions on growth performance, energy utilization, and mineral digestibility.

The present study addressed this knowledge gap using a deliberately P-restricted corn–soybean meal-based grower diet containing 0.40% total P, 0.26% phytate P, and 0.14% non-phytate P, without supplemental inorganic P. This nutritional model provides an opportunity to evaluate the intrinsic responses to phytase and β-mannanase under conditions in which dietary P availability is strongly constrained. Determining the individual and interactive responses to these enzymes may also have practical implications for optimizing enzyme supplementation, reducing dependence on inorganic P sources, improving utilization of dietary P, and potentially reducing P excretion associated with broiler production.

This study aimed to evaluate the individual and interactive effects of dietary phytase and β-mannanase supplementation on growth performance, ileal digestible energy (IDE), and apparent ileal Ca and P digestibility in Ross 308 broilers fed a P-deficient diet without supplemental inorganic P. Specifically, the study evaluated whether phytase supplementation improved growth performance and mineral utilization, whether β-mannanase supplementation enhanced energy and nutrient utilization, and whether simultaneous supplementation with both enzymes produced interactive effects under P-restricted dietary conditions. It was hypothesized that phytase would primarily improve P and Ca utilization, whereas β-mannanase would primarily enhance energy utilization and nutrient accessibility, with combined supplementation potentially producing additive or synergistic responses.

## MATERIALS AND METHODS

### Ethical approval

All procedures involving animals were reviewed and approved by the Kasetsart University Institutional Animal Care and Use Committee (Approval No. ACKU68-AGR-006). Animal care, housing, experimental procedures, sample collection, and euthanasia were conducted in accordance with the guidelines of the National Research Council of Thailand. Throughout the experimental period, birds were maintained under environmentally controlled conditions, with temperature and lighting managed according to the Ross 308 Broiler Management Handbook. On day 24, birds selected for ileal digesta collection were euthanized by cervical dislocation before sample collection.

### Study period and location

The experiment was conducted from February to March 2025 at the Department of Animal Science, Faculty of Agriculture, Kasetsart University, Bangkhen, Bangkok, Thailand. A total of 720 one-day-old Ross 308 male broiler chicks obtained from a commercial hatchery were reared for 24 days in an environmentally controlled poultry house equipped with an evaporative cooling system. Temperature and lighting were managed according to the Ross 308 Broiler Management Handbook.

### Study design

A total of 720 broilers were randomly allocated to four dietary treatments in a completely randomized design with a 2 × 2 factorial arrangement. The two experimental factors were phytase supplementation at 0 or 1,000 FTU/kg and β-mannanase supplementation at 0 or 800 Thermostable mannanase unit (TMU)/kg. The four treatments were T1, control diet without phytase or β-mannanase; T2, β-mannanase at 800 TMU/kg; T3, phytase at 1,000 FTU/kg; and T4, phytase at 1,000 FTU/kg plus β-mannanase at 800 TMU/kg. Each treatment consisted of 12 replicate pens, with 15 birds per pen and a stocking density of 12.5 birds/m². Thus, the experiment comprised 48 pens, with the pen serving as the experimental unit. The factorial design was used to evaluate the main effects of phytase and β-mannanase and their interaction.

### Diets and feeding management

All birds received a common starter diet from days 1 to 7 that was formulated to meet the nutritional recommendations for Ross 308 broilers. From days 8 to 24, birds received experimental grower diets formulated primarily with corn and soybean meal, following the recommendations of Leeson and Summers [9]. Diet formulation was based on the analyzed nutrient composition of the raw materials (Tables 1 and 2).

The experimental grower diet was deliberately formulated without supplemental inorganic P to evaluate the responses to phytase and β-mannanase under P-restricted conditions. The diet contained 0.40% total P, 0.14% non-phytate P, and 0.26% phytate P. Phytase and β-mannanase were incorporated at 1,000 FTU/kg and 800 TMU/kg, respectively, according to treatment allocation. The four treatments therefore represented diets without either enzyme, with β-mannanase alone, with phytase alone, or with both enzymes.

The phytase preparation was Natuphos E 10000 G derived from *Aspergillus niger* (BASF SE, Ludwigshafen am Rhein, Germany), and the β-mannanase preparation was Natupulse TS derived from *Thermothelomyces thermophilus* (BASF SE, Ludwigshafen am Rhein, Germany). Phytase activities in the phytase-supplemented diets were 1,201 FTU/kg and 1,278 FTU/kg for treatments T3 and T4, respectively. The corresponding analyzed β-mannanase activities were 853 TMU/kg and 858 TMU/kg for treatments T2 and T4, respectively. Phytase activity was determined according to ISO 30024:2024, whereas β-mannanase activity was determined using the BASF in-house analytical method.

Experimental diets were pelleted through a 3.0-mm die at a conditioning temperature of 65°C–70°C. Feed and water were provided *ad libitum* throughout the experimental period using tube feeders and nipple drinkers.

**Table 1:** Ingredient composition of the experimental grower diet.

**Ingredient**	**Grower diet (days 8–24), %**
Maize	58.37
Soybean meal (48% crude protein)	31.01
Palm oil	4.53
Rapeseed meal	4.18
Limestone powder	1.11
Sodium bicarbonate	0.25
Salt	0.27
Choline chloride (60%)	0.04
Vitamin-mineral premixᵃ	0.25
Total	100.00

ᵃVitamin-mineral premix provided per kilogram of diet: vitamin A palmitate = 5,850 IU; vitamin D₃ = 2,700 IU; vitamin E acetate = 27 IU; vitamin K = 1.8 mg; riboflavin = 5 mg; niacin = 36 mg; calcium pantothenate = 12.6 mg; vitamin B₁₂ = 0.012 mg; iron = 80 mg; iodine = 0.40 mg; manganese = 70 mg; selenium = 0.30 mg.

ᵇThe analyzed phytase activities in the phytase-supplemented diets were 1,201 FTU/kg (T3) and 1,278 FTU/kg (T4), whereas the analyzed β-mannanase activities in the β-mannanase-supplemented diets were 853 TMU/kg (T2) and 858 TMU/kg (T4), as determined according to ISO 30024:2024 and the BASF in-house analytical method, respectively.

FTU = Phytase unit; TMU = Thermostable mannanase unit.

**Table 2:** Nutrient composition of the experimental grower diet.

**Nutrient**	**Unit**	**Grower diet (days 8–24)**
Moisture	%	11.04
AMEn	kcal/kg	3,000.00
Crude protein	%	20.64
Lysine	%	1.11
Methionine	%	0.31
Cysteine	%	0.34
Methionine + cysteine	%	0.65
Arginine	%	1.37
Threonine	%	0.79
Isoleucine	%	0.88
Leucine	%	1.72
Valine	%	0.98
Ether extract	%	7.55
Crude fiber	%	2.90
ADF	%	3.73
NDF	%	9.09
Starch	%	38.25
Sugars	%	3.27
Ca	%	0.60
Total P	%	0.40
Non-phytate P	%	0.14
Phytate P	%	0.26

^a^AMEn = Nitrogen-corrected apparent metabolizable energy; ADF = Acid detergent fiber; Ca = Calcium; NDF = Neutral detergent fiber; P = Phosphorus.

^b^Nutrient values presented in Table 2 are calculated/formulated values based on the analyzed nutrient composition of the raw materials, including moisture, crude protein, ether extract, crude fiber, Ca, P, ADF, NDF, total sugars, and starch.

### Growth performance measurements

Body weight (BW) was recorded on days 8 and 24, and the values were used to calculate average daily gain (ADG) during the grower period. Feed intake (FI) was determined as the difference between the amount of feed offered and the feed remaining. Feed conversion ratio (FCR) was calculated as FI divided by BW gain. Mortality was recorded daily and used to adjust growth performance calculations where appropriate.

### Ileal digesta collection and digestibility determination

IDE and Apparent ileal digestibility (AID) of Ca and P were determined using Celite as an indigestible marker, which was added on top of the formulated diets at 1% (Celite 545, Sigma-Aldrich, St. Louis, MO, USA). On day 24, one bird from each pen was selected and euthanized by cervical dislocation, resulting in 12 birds per treatment and 48 birds overall. Ileal digesta were collected from the intestinal segment extending from Meckel's diverticulum to approximately 40 mm anterior to the ileocecal junction following the sampling procedure described by Huang *et al.* [10].

Ileal digesta were gently expressed from the intestinal segment into collection containers and immediately stored at −20°C. The samples were subsequently transferred to −80°C until further processing. Before chemical analysis, digesta samples were freeze-dried under vacuum conditions using a VirTis freeze dryer (SP Scientific, USA). Diet and digesta samples were analyzed for gross energy using an adiabatic bomb calorimeter and for Ca and P according to Association of Official Analytical Chemists procedures [11]. Acid-insoluble ash (AIA) concentrations in the diet and ileal digesta were determined after ashing and treatment with boiling 4 M HCl, followed by ultraviolet-visible spectrophotometry. All laboratory analyses were performed in duplicate.

AID was calculated using the standard marker-ratio method [12]:

AID (%) = 100 − [100 × (AIA in diet / AIA in ileal digesta) × (Nutrient in ileal digesta / Nutrient in diet)]. 

IDE was calculated using the following equation:

IDE (kcal/kg) = GE in diet − [GE in ileal digesta × (AIA in diet / AIA in ileal digesta)], 

where AIA represents acid-insoluble ash and GE represents gross energy.

### Statistical analysis

All data were analyzed using SAS Studio (SAS Institute Inc., Cary, NC, USA). The pen was considered the experimental unit for all measurements. Data were analyzed as a completely randomized design with a 2 × 2 factorial arrangement, including the main effects of β-mannanase and phytase and their interaction. The statistical model was as follows:

Yijk​=μ+Mi​+Pj​+(MP)ij​+eijk​

where Yijk = dependent variable; μ = overall mean; Mi = main effect of β-mannanase; Pj = main effect of phytase; (MP)ij = interaction between β-mannanase and phytase; and eijk = random error term. 

When significant treatment effects were detected, means were separated using Duncan's multiple range test [13]. Statistical significance was set at p ≤ 0.05.

## RESULTS

### Growth performance

Growth performance during the experimental period from days 8 to 24 is presented in Table 3. No mortality was recorded during the experimental period. Flock uniformity ranged from 86.07% to 88.62% across dietary treatments. Neither β-mannanase nor phytase had a significant main effect on flock uniformity (p > 0.05); however, a significant β-mannanase × phytase interaction was observed (p < 0.05).

β-Mannanase supplementation had no significant main effect on final BW, ADG, FI, or FCR (p > 0.05). In contrast, phytase supplementation significantly increased final BW from 1,093.11 to 1,251.03 g (14.4%), ADG from 52.12 to 61.40 g/day (17.8%), and FI from 72.19 to 82.59 g/day (14.4%) and improved FCR from 1.39 to 1.35 (p < 0.01). No significant β-mannanase × phytase interactions were observed for final BW, ADG, FI, or FCR (p > 0.05).

**Table 3:** Effects of β-mannanase and phytase supplementation on growth performance of broilers from days 8 to 24.

**Item**		**Control**		**β-** **Mannanase**		**Phytase**		**β-** **Mannanase** ** + phytase**			**p-value**		**SEM**
Initial BW (g)		207.43 ± 21.93		206.83 ± 20.38		207.37 ± 23.83		207.02 ± 22.33			0.99		0.82
Final BW (g)		1,104.36 ± 136.81ᵇ		1,081.86 ± 156.99ᵇ		1,248.56 ± 170.94ᵃ		1,253.27 ± 151.58ᵃ			<0.01		6.47
ADG (g/day)		52.76 ± 2.19ᵇ		51.47 ± 3.15ᵇ		61.26 ± 2.97ᵃ		61.54 ± 3.39ᵃ			<0.01		0.80
FI (g/day)		72.85 ± 2.51ᵇ		71.53 ± 3.23ᵇ		83.04 ± 3.52ᵃ		82.13 ± 3.78ᵃ			<0.01		0.89
FCR		1.38 ± 0.03ᵃ		1.39 ± 0.04ᵃ		1.36 ± 0.04ᵃᵇ		1.34 ± 0.05ᵇ			<0.05		0.01
Uniformity (%)		88.02 ± 2.68		86.07 ± 1.95		86.86 ± 3.09		88.62 ± 1.74			0.06		0.70
**Main effects**													
**Item**	**β-** **Mannanase** ** −**		**β-** **Mannanase** ** +**		**Phytase −**		**Phytase +**		**p-value M**	**p-value P**		**p-value M × P**	
Final BW (g)	1,176.58 ± 87.83		1,167.56 ± 105.35		1,093.11 ± 49.97ᵇ		1,251.03 ± 57.84ᵃ		0.57	<0.01		0.40	
ADG (g/day)	57.01 ± 5.04		56.51 ± 6.06		52.12 ± 2.74ᵇ		61.40 ± 3.12ᵃ		0.56	<0.01		0.36	
FI (g/day)	77.95 ± 6.01		76.83 ± 6.41		72.19 ± 2.91ᵇ		82.59 ± 3.60ᵃ		0.25	<0.01		0.83	
FCR	1.37 ± 0.04		1.36 ± 0.06		1.39 ± 0.04ᵃ		1.35 ± 0.05ᵇ		0.68	<0.01		0.23	
Uniformity (%)	87.44 ± 2.89		87.35 ± 2.23		87.05 ± 2.61		87.74 ± 2.61		0.89	0.33		0.011	

ᵃᵇMeans with different superscripts within the same row differ significantly (p < 0.05). Values are presented as mean ± standard deviation. ADG = Average daily gain; BW = Body weight; FCR = Feed conversion ratio; FI = Feed intake; FTU = Phytase unit; M = Main effect of β-mannanase; P = Main effect of phytase; SEM = Standard error of the mean; TMU = Thermostable mannanase unit. Phytase and β-mannanase were supplemented at 1,000 FTU/kg and 800 TMU/kg, respectively. M × P = β-Mannanase × phytase interaction.

### IDE and mineral digestibility

The effects of β-mannanase and phytase supplementation on IDE and apparent ileal Ca and P digestibility on day 24 are presented in Table 4. Compared with the control diet, all enzyme-supplemented treatments had significantly higher IDE (p < 0.01), with no significant differences among the β-mannanase, phytase, and combined-enzyme treatments. Factorial analysis showed that β-mannanase supplementation increased IDE from 2,647.01 to 2,681.13 kcal/kg, whereas phytase supplementation increased IDE from 2,641.84 to 2,686.30 kcal/kg (p < 0.01). No significant β-mannanase × phytase interaction was observed for IDE (p > 0.05).

Apparent ileal Ca digestibility was significantly higher in birds supplemented with phytase alone or in combination with β-mannanase than in birds receiving the control diet (p < 0.01), whereas β-mannanase alone resulted in an intermediate value. Factorial analysis showed that β-mannanase increased apparent ileal Ca digestibility from 58.00% to 58.70% (p < 0.05), whereas phytase increased it from 57.82% to 58.83% (p < 0.01). No significant interaction between β-mannanase and phytase was detected for apparent ileal Ca digestibility (p > 0.05).

Apparent ileal P digestibility was significantly higher in birds receiving phytase alone or the combined-enzyme treatment than in those receiving the control diet (p < 0.05), whereas β-mannanase alone produced an intermediate response. Factorial analysis showed no significant main effect of β-mannanase on apparent ileal P digestibility (p > 0.05). In contrast, phytase supplementation increased apparent ileal P digestibility by 3.24 percentage points, from 71.59% to 74.83% (p < 0.01). No significant β-mannanase × phytase interaction was detected for apparent ileal P digestibility (p > 0.05).

**Table 4:** Effects of β-mannanase and phytase supplementation on ileal digestible energy and apparent ileal calcium and phosphorus digestibility in broilers on day 24.

**Item**		**Control**		**β-** **Mannanase**		**Phytase**		**β-** **Mannanase** ** + phytase**			**p-value**		**SEM**
IDE (kcal/kg)		2,618.24 ± 21.05ᵇ		2,665.43 ± 51.46ᵃ		2,675.78 ± 49.50ᵃ		2,696.83 ± 9.04ᵃ			<0.01		7.01
Apparent ileal Ca digestibility (%)		57.41 ± 0.40ᵇ		58.24 ± 1.39ᵃᵇ		58.54 ± 1.51ᵃ		59.13 ± 0.21ᵃ			<0.01		0.18
Apparent ileal P digestibility (%)		70.64 ± 3.27ᵇ		72.54 ± 2.47ᵃᵇ		75.35 ± 3.95ᵃ		74.30 ± 2.13ᵃ			<0.05		0.57
**Main effects**													
**Item**	**β-** **Mannanase** ** −**		**β-** **Mannanase** ** +**		**Phytase −**		**Phytase +**		**p-value M**	**p-value P**		**p-value ** **M × P**	
IDE (kcal/kg)	2,647.01 ± 47.48ᵇ		2,681.13 ± 39.47ᵃ		2,641.84 ± 45.44ᵇ		2,686.30 ± 36.35ᵃ		<0.01	<0.01		0.26	
Apparent ileal Ca digestibility (%)	58.00 ± 1.24ᵇ		58.70 ± 1.05ᵃ		57.82 ± 1.08ᵇ		58.83 ± 1.09ᵃ		<0.05	<0.01		0.71	
Apparent ileal P digestibility (%)	73.00 ± 4.27		73.42 ± 2.41		71.59 ± 2.97ᵇ		74.83 ± 3.12ᵃ		0.68	<0.01		0.16	

ᵃᵇMeans with different superscripts within the same row differ significantly (p < 0.05). Values are presented as mean ± standard deviation. Ca = Calcium; FTU = Phytase unit; IDE = Ileal digestible energy; Treatment p-value represents the overall comparison among the four dietary treatments; M = Main effect of β-mannanase; P = Main effect of phytase; SEM = Standard error of the mean; TMU = Thermostable mannanase unit. Phytase and β-mannanase were supplemented at 1,000 FTU/kg and 800 TMU/kg, respectively. M × P = β-Mannanase × phytase interaction.

## DISCUSSION

The present study provides evidence that phytase and β-mannanase produced largely independent responses in broilers fed a P-deficient diet without supplemental inorganic P. Phytase markedly improved growth performance, IDE, and apparent ileal Ca and P digestibility, whereas β-mannanase primarily increased IDE and produced a modest improvement in apparent ileal Ca digestibility. No significant β-mannanase × phytase interactions were detected for growth performance, IDE, or mineral digestibility. This response pattern differs from some previous studies reporting additive or synergistic responses under less restrictive dietary conditions and suggests that dietary nutrient constraints may influence the magnitude and interaction of responses to exogenous enzymes.

### Growth performance

Phytase supplementation significantly increased final BW, ADG, and FI and improved FCR, consistent with previous studies reporting improved growth performance and nutrient utilization following phytate degradation [14, 15]. In the present study, the grower diet contained no supplemental inorganic P and provided only 0.14% non-phytate P. Under these conditions, phytate-bound P represented an important dietary P source, providing a plausible explanation for the pronounced growth response to phytase supplementation. Hydrolysis of phytate by phytase can increase the availability of phytate-bound P and reduce the anti-nutritional effects associated with phytate–nutrient interactions, thereby improving nutrient utilization.

In contrast, β-mannanase supplementation did not significantly affect final BW, ADG, FI, or FCR. Although β-mannanase can improve nutrient utilization by hydrolyzing β-mannan in plant-based feed ingredients, the lack of a growth response in the present study suggests that its effects on nutrient utilization were insufficient to translate into measurable performance improvements under the experimental conditions. The strong response to phytase relative to β-mannanase may indicate that P availability represented a more important nutritional constraint than the substrates targeted by β-mannanase. However, because the relative contributions of individual nutrient limitations were not directly quantified, interpret this explanation cautiously.

A significant β-mannanase × phytase interaction was detected for flock uniformity, despite the absence of significant main effects of either enzyme. The interaction should be interpreted cautiously because no corresponding interactions were detected for final BW, ADG, FI, or FCR. Furthermore, the overall treatment comparison for flock uniformity was not significant. Thus, the observed interaction does not provide sufficient evidence of a consistent complementary effect of the two enzymes on growth performance.

## IDE

Both β-mannanase and phytase independently increased IDE, indicating that the two enzymes influenced energy utilization despite their different primary substrates. The increase in IDE associated with β-mannanase supplementation is consistent with previous reports describing improved nutrient utilization following β-mannan hydrolysis [8, 16]. β-Mannanase has been proposed to modify β-mannan-containing cell-wall structures and the matrix surrounding protein storage vacuoles, potentially increasing nutrient accessibility within soybean meal [17]. These effects may improve energy utilization even when measurable improvements in growth performance are not observed.

Phytase also significantly increased IDE. In addition to binding P and other minerals, phytate can interact with dietary proteins and influence nutrient digestion. Hydrolysis of phytate may therefore alleviate some of these anti-nutritional effects and improve the utilization of dietary energy and other nutrients. This interpretation is consistent with the anti-nutritional effects of phytate described by Selle *et al.* [1]. However, the present study did not directly measure amino acid digestibility or endogenous nutrient losses; therefore, these mechanisms cannot be confirmed from the current findings.

Importantly, no significant β-mannanase × phytase interaction was detected for IDE. Thus, although both enzymes significantly increased IDE, their simultaneous supplementation did not produce a statistically demonstrable synergistic response under the conditions evaluated.

### Calcium and P digestibility

Phytase supplementation significantly increased apparent ileal Ca and P digestibility, supporting its established role in hydrolyzing phytate and increasing the availability of phytate-associated minerals [2, 5]. These findings are consistent with those of Venter *et al.* [18], who reported improved Ca and P digestibility following phytase supplementation across diets differing in phytate P content. The response observed in the present study is particularly relevant because the experimental diet contained no supplemental inorganic P, increasing the nutritional importance of P released from dietary phytate.

β-Mannanase also produced a significant, although relatively modest, increase in apparent ileal Ca digestibility, from 58.00% to 58.70%. In contrast, β-mannanase did not significantly affect apparent ileal P digestibility. Modification of β-mannan-containing feed structures could potentially improve mineral accessibility within the gastrointestinal tract. However, the biological mechanism underlying the observed Ca response cannot be established from the present experiment. Digesta viscosity, mineral solubilization, endogenous Ca losses, and other potential mechanisms were not measured; therefore, mechanistic explanations for this finding remain speculative.

Phytase increased apparent ileal P digestibility from 71.59% to 74.83%, representing an increase of 3.24 percentage points. This response, together with improved growth performance, demonstrates that phytase was particularly effective under the P-restricted conditions evaluated. Nevertheless, AID represents nutrient disappearance before the terminal ileum and does not directly quantify whole-body P retention or bone mineralization. Consequently, the present findings should not be interpreted as evidence that phytase completely satisfied the birds' physiological P requirements.

### Interaction between phytase and β-mannanase

No significant β-mannanase × phytase interactions were detected for final BW, ADG, FI, FCR, IDE, or apparent ileal Ca and P digestibility. This finding contrasts with previous studies reporting additive or synergistic responses to enzyme combinations in reduced-energy or reduced-protein diets [19, 20]. Differences in dietary formulation may partly explain these contrasting responses because enzyme efficacy depends on substrate availability, nutrient concentrations, and the nutritional constraints imposed by the basal diet.

In the present study, complete omission of supplemental inorganic P created a dietary condition in which the availability of phytate-bound P was particularly important. The pronounced response to phytase suggests that alleviation of phytate-associated nutrient constraints had a greater influence on growth performance than β-mannan hydrolysis. Under these conditions, the scope for β-mannanase to further improve growth performance may have been limited. Nevertheless, describing this response as a “ceiling effect” would require additional experimental evidence across multiple dietary P concentrations. The present findings establish the absence of statistically significant interactions under the conditions tested but do not demonstrate the mechanism responsible for that absence.

### Study limitations and future research

This study had several limitations. First, the experiment was restricted to the grower period (days 8 to 24) and evaluated only one supplementation level of phytase and β-mannanase under a single P-deficient dietary condition. Consequently, dose-dependent responses and enzyme interactions across different dietary P concentrations could not be evaluated. Second, digesta viscosity was not measured, limiting direct assessment of a mechanism potentially associated with β-mannanase supplementation. Third, intestinal microbial composition was not evaluated; therefore, potential effects of enzyme supplementation on the intestinal microbiota could not be determined.

Furthermore, tibia ash and other indices of bone mineralization were not evaluated. The findings regarding Ca and P utilization are therefore limited to AID and cannot establish effects on whole-body mineral retention or skeletal mineralization. Although nutrient profiles of the raw materials were analyzed before diet formulation, responses to enzyme supplementation may differ with ingredient composition and practical feeding conditions. Future studies should evaluate multiple phytase and β-mannanase inclusion levels across graded dietary P concentrations and longer production phases. Incorporating measurements of bone mineralization, nutrient retention, digesta characteristics, and other mechanistic indicators would further clarify the nutritional consequences and potential interactions of these enzymes.

### Practical implications

The present findings demonstrate that phytase has an important nutritional role in broiler grower diets formulated without supplemental inorganic P, as reflected by improvements in growth performance, IDE, and apparent ileal Ca and P digestibility. β-Mannanase provided additional, independent responses in IDE and apparent ileal Ca digestibility but did not significantly improve growth performance or P digestibility. These findings highlight the distinct nutritional responses associated with the two enzymes and indicate that their combined effects depend on the dietary conditions under which they are applied.

From a practical perspective, the pronounced phytase response indicates the potential to substantially reduce reliance on supplemental inorganic P in carefully formulated broiler diets. Improved utilization of dietary P could potentially reduce P excretion and decrease dependence on inorganic phosphate sources. Similarly, the improvement in IDE associated with β-mannanase may provide opportunities for more efficient utilization of dietary energy. However, the present study did not directly evaluate feed cost, P excretion, nutrient retention, or environmental outcomes. Therefore, dedicated studies should confirm economic and environmental benefits. In addition, longer-term and multi-phase studies under commercial production conditions, including assessment of bone mineralization and bird performance through market age, are required before complete elimination of supplemental inorganic P can be broadly recommended.

## CONCLUSION

The present study demonstrated that phytase and β-mannanase produced distinct and largely independent nutritional responses in Ross 308 broilers fed a P-deficient grower diet without supplemental inorganic P. Phytase supplementation increased final BW from 1,093.11 to 1,251.03 g, ADG from 52.12 to 61.40 g/day, and FI from 72.19 to 82.59 g/day and improved FCR from 1.39 to 1.35. Phytase also increased IDE from 2,641.84 to 2,686.30 kcal/kg and apparent ileal Ca and P digestibility from 57.82% to 58.83% and from 71.59% to 74.83%, respectively. β-Mannanase did not significantly affect growth performance or apparent ileal P digestibility but increased IDE from 2,647.01 to 2,681.13 kcal/kg and apparent ileal Ca digestibility from 58.00% to 58.70%. No significant β-mannanase × phytase interactions were detected for growth performance, IDE, or apparent ileal Ca and P digestibility, although a significant interaction was observed for flock uniformity.

A major strength of this study was its controlled 2 × 2 factorial design, which allowed the individual effects of phytase and β-mannanase to be distinguished from their potential interaction. The experimental model was also characterized by the complete omission of supplemental inorganic P, with the grower diet containing 0.40% total P, 0.14% non-phytate P, and 0.26% phytate P. This design provided a stringent nutritional setting for evaluating enzyme responses when dietary P availability was substantially constrained. Furthermore, the study simultaneously evaluated growth performance, IDE, and apparent ileal Ca and P digestibility, allowing characterization of the two enzymes’ responses across complementary performance and nutrient-utilization outcomes.

Overall, phytase was the principal enzyme associated with improvements in growth performance and mineral utilization under the P-restricted conditions evaluated, whereas β-mannanase primarily improved energy utilization and produced a modest increase in apparent ileal Ca digestibility. The lack of significant interactions for the major growth performance and nutrient utilization outcomes indicates that combining phytase and β-mannanase did not produce a detectable synergistic response under the experimental conditions. Collectively, these findings show that nutritional responses to phytase and β-mannanase differ according to their respective functional roles and provide evidence of largely independent effects when broilers are fed a grower diet without supplemental inorganic P.

## DATA AVAILABILITY

The datasets of this study are available from the corresponding author upon reasonable request.

## GENERATIVE AI DECLARATION

The authors declare that they used generative artificial intelligence (AI) tools solely to improve language, grammar, and readability during manuscript preparation. All scientific content, data analysis, interpretation of results, and conclusions were developed and verified by the authors. The authors take full responsibility for the accuracy, integrity, and originality of the work presented, and no AI tool was listed as an author.

## AUTHORS’ CONTRIBUTIONS

TP: Conceptualization, study design, investigation, statistical analysis, and writing – original draft. TPo: Conceptualization, supervision, and writing – review and editing. CB, CR, and SP: Supervision, interpretation of results, and writing – review and editing. All authors have read and approved the final version of the manuscript.
